# Impact of Influenza A Virus Shutoff Proteins on Host Immune Responses

**DOI:** 10.3390/vaccines9060629

**Published:** 2021-06-10

**Authors:** Megan M. Dunagan, Kala Hardy, Toru Takimoto

**Affiliations:** Department of Microbiology and Immunology, University of Rochester Medical Center, 601 Elmwood Ave., Rochester, NY 14642, USA; Megan_Dunagan@urmc.rochester.edu (M.M.D.); Kala_Harbaugh@urmc.rochester.edu (K.H.)

**Keywords:** influenza A virus, host shutoff, NS1, PA-X

## Abstract

Influenza A virus (IAV) is a significant human pathogen that causes seasonal epidemics. Although various types of vaccines are available, IAVs still circulate among human populations, possibly due to their ability to circumvent host immune responses. IAV expresses two host shutoff proteins, PA-X and NS1, which antagonize the host innate immune response. By transcriptomic analysis, we previously showed that PA-X is a major contributor for general shutoff, while shutoff active NS1 specifically inhibits the expression of host cytokines, MHC molecules, and genes involved in innate immunity in cultured human cells. So far, the impact of these shutoff proteins in the acquired immune response in vivo has not been determined in detail. In this study, we analyzed the effects of PA-X and NS1 shutoff activities on immune response using recombinant influenza A/California/04/2009 viruses containing mutations affecting the expression of shutoff active PA-X and NS1 in a mouse model. Our data indicate that the virus without shutoff activities induced the strongest T and B cell responses. Both PA-X and NS1 reduced host immune responses, but shutoff active NS1 most effectively suppressed lymphocyte migration to the lungs, antibody production, and the generation of IAV specific CD4^+^ and CD8^+^ T cells. NS1 also prevented the generation of protective immunity against a heterologous virus challenge. These data indicate that shutoff active NS1 plays a major role in suppressing host immune responses against IAV infection.

## 1. Introduction

Seasonal influenza A virus (IAV) infection is a major public health problem causing 3–5 million hospitalizations and 50,000 deaths annually in the USA [1]. In addition to the endemic H3N2 and H1N1 strains, the wide array of antigenically distinct IAVs circulating in avian and other zoonotic reservoirs pose a pandemic risk as a source of genomic reassortment. Even though various types of vaccines are available, it is hard to control seasonal IAV epidemics. Moreover, repeated infection occurs within the population, suggesting that IAV can circumvent anti-IAV immunity or that natural infection induces insufficient immunity to prevent repeated infection. Studies on various human pathogens indicate that many viruses express accessory proteins to modulate host innate and acquired immune responses [2]. Some viruses express proteins which induce a general shutoff of host protein synthesis [3]. In the case of IAV, two viral proteins, NS1 and PA-X, have host shutoff functions and play a major role in regulating host gene expression to block antiviral activities [4,5,6,7,8].

NS1 is a multifunctional protein expressed from the NS gene segment with a strain-specific size of 230–237 amino acids [6,9]. NS1 is one of the best characterized viral proteins and has been shown to have multiple functions, such as innate immune evasion activity and limiting antiviral activity of double-stranded RNA-dependent protein kinase R (PKR) and 2′5′-oligoadenylate synthetase (OAS)/RNase L [6]. Additionally, IAV NS1 suppresses host pre-mRNA maturation through its association with a subunit of the cleavage and polyadenylation specificity factor 30 (CPSF30) [10,11,12]. Crystal structures show an association between the effector domain of CPSF30 (F2-F3 zinc fingers) and NS1 residues F103, M106, K108, D125, and D189 [10]. These residues are highly conserved among seasonal human isolates. However, the 2009 pandemic H1N1 (2009pH1N1) virus derived from swine hosts contain mutations in its CPSF30-binding region at K108R, D125E, and D189G, preventing NS1-CPSF30 association and NS1-mediated host shutoff [6,13]. Mutations at these three residues back to the conserved residues restore the host shutoff activity [13,14]. 

Another IAV host shutoff protein is PA-X, which is expressed by a ribosomal frameshift during translation of the viral polymerase subunit PA mRNA [5]. PA-X shares its first 191 amino acids with PA and contains a unique 41 or 61 amino acid C-terminal sequence depending on the strain [15]. Due to the low frequency of frameshift events, very little PA-X is expressed in infected cells, but its shutoff activity far exceeds that of NS1 as determined in vitro [14]. Host shutoff activity of PA-X is linked to its endonuclease activity within the N-terminal domain [5,14,16]. Although the mechanism of how PA-X targets host mRNAs is not fully elucidated, PA-X specifically targets RNAs transcribed by host RNA polymerase II, suggesting its specific interaction with host transcription machinery [17]. Like NS1, shutoff activity of PA-X varies between virus isolates. PA-X of seasonal human IAV isolates is less active than that of avian IAVs, likely due to mutations within the N-terminal domain shared with PA [16,18,19]. Previous studies on IAV shutoff activities suggest a functional interplay between the two proteins in a species-specific manner that is likely adjusted to achieve optimal infectivity within specific hosts [4]. 

To analyze the target specificity and functional role of IAV shutoff proteins, we created mutant viruses using a 2009pH1N1 virus, A/California/04/2009 (Cal), as a backbone [4,8]. We mutated the NS gene to gain the shutoff function or the PA gene to suppress frameshift efficiency. Through the combination of these mutations, we were able to rescue Cal viruses expressing shutoff active NS1 and PA-X in various combinations. Our in vitro analysis in human airway A549 cells indicate that PA-X is the major factor for general shutoff, while shutoff active NS1 allows specific suppression of genes involved in innate and acquired immune responses [4]. However, the actual impact of these shutoff proteins in host immune responses in vivo has not been determined. In this study, we investigated the effects of PA-X and NS1 on viral virulence, immune infiltration to the respiratory tract, generation of IAV-specific T and B cells, and establishment of protective immunity against subsequent challenge with homologous and heterologous IAVs. Our data indicate that both shutoff proteins suppress host immune responses, while shutoff active NS1 plays a critical role in inhibiting the establishment of long-lasting immunity against IAV. 

## 2. Materials and Methods

### 2.1. Viruses and Cells 

Influenza A virus A/California/04/2009 (H1N1) was provided by R. Webster and R. Webby (St. Jude Children’s Research Hospital, Memphis, TN, USA). Stock virus was propagated in 10-day-old embryonated chicken eggs. A/Puerto Rico/8/34 (H1N1) was provided by S. Dewhurst (University of Rochester, Rochester, NY, USA) and grown in MDCK cells. Rescue of the mutant viruses were reported previously [4,8,20]. Virus titer was measured by plaque assay in MDCK cells. MDCK cells were maintained in Dulbecco’s modified Eagle’s medium (DMEM; Corning, Glendale, AZ, USA) supplemented with 8% fetal bovine serum (FBS, VWR/Seradigm, Radnor, PA, USA) and gentamicin (Thermo Fisher Scientific, Waltham, MA, USA).

### 2.2. Animals

Six to eight-week-old specific-pathogen free C57BL/6 female mice were purchased from The Jackson Laboratory (Mount Desert Island, ME, USA). All animals were housed in the University of Rochester Vivarium facilities under specific pathogen-free conditions using microisolator technology. All animal protocols and group numbers were approved by the University of Rochester Committee of Animal Resources and complied with the recommendations in the Guide for the Care and Use of Laboratory Animals of the National Research Council.

### 2.3. Virus Growth and Pathogenicity 

To determine viral growth, mice were anesthetized with Avertin (240 mg/kg of body weight) and mock infected with PBS or infected with wild type (wt) or mutant Cal viruses at a dose of 10^3^ PFU intranasally. At 1, 3, 5, and 7 days post infection (dpi), groups of 4 mice were humanely sacrificed. Whole lungs were collected and homogenized in 1 mL of DMEM containing gentamicin (50 μg/mL). Viral titers in clarified supernatants were determined using MDCK cells and 50% cell culture infectious doses (TCID_50_) were calculated by the Reed–Muench method [21]. To determine the 50% median lethal dose (MLD_50_) of the viruses, groups of 5 mice were infected intranasally with wt or mutant Cal viruses at 10-fold serial dilutions containing 10^2^ to 10^4^ PFU in a 20 μL volume. The body weight of each individual mouse was measured daily for 15 days. Thirty percent weight loss was considered fatal, and mice reaching this limit were humanely sacrificed. The MLD_50_ of each virus was also calculated by the Reed–Muench method.

### 2.4. Infiltration of Immune Cells into the Respiratory Tract 

Bronchoalveolar lavage fluid (BALF) was collected from the lungs of mice mock infected or infected with 1000 pfu of the viruses as described previously [22]. Briefly, a cannula was inserted into the trachea and 0.8 mL of PBS was injected and aspirated 3 times. This process was repeated 3 times. Collected fluids were centrifuged at 800× *g* for 10 min at 4 °C. The soluble fraction of the first lavage was saved for ELISA analysis. Cellular components of the 3 washes were combined, counted, and affixed to microscope slides using centrifugation. Cells were then fixed and stained using the Hema3 fixative and staining kit (Thermo Fisher Scientific, Waltham, MA, USA). Cellular infiltrates were identified by morphology on 1, 3, 5, 7, 21 and 84 days post infection [23]. 

### 2.5. ELISA for Cytokines and Antibodies 

BALF was collected as above and IL-6 and IFN-γ were quantitated using commercially available kits (Mouse IL-6 Quantikine ELISA Kit and Mouse IFN-gamma Quantikine ELISA Kit, R&D Systems, Minneapolis, MN, USA). HA-specific serum antibodies at 21 and 84 dpi were detected by chemiluminescent ELISA. Purified Cal HA (NR-15749; BEI Resources, Manassas, VA, USA) was coated on a 96-well plate overnight at 4 °C (0.1 μg/well). Plates were blocked with 1% BSA-PBS for 1 h and then incubated with serial dilutions of sera collected from infected or mock-infected mice for 1 h at 37 °C. The plate was washed and incubated with HRP-conjugated goat anti-mouse IgG (Bio-Rad, Hercules, CA, USA) for 1 h at 37 °C. Reactions were developed using SuperSignal™ West Femto Maximum Sensitivity Substrate (Thermo Fisher Scientific, Waltham, MA, USA) and measured by a DTX 880 Multimode Detector (Beckman Coulter, Indianapolis, IN, USA). Samples were run in duplicate.

### 2.6. ELISpot Assays for T and B Cells 

T cell ELISpot assays were performed as described previously [24]. Single cell suspensions were made from mediastinal lymph nodes (MLN) and spleens of virus-infected or mock-infected animals at 7, 21 and 84 dpi and processed in DMEM supplemented with 8% FBS. Red blood cells were depleted by treatment with red blood cell lysis buffer (0.15 M NH_4_Cl, 1 mM KHCO_3_, 0.1 mM Na_2_EDTA in H_2_O, pH 7.2 to 7.4) for 5 min at room temperature. Washed cells were enriched using negative selection kits for either CD4^+^ (Dynabeads™ Untouched™ Mouse CD^4^ Cells Kit, Thermo Fisher Scientific, Waltham, MA, USA) or CD8^+^ (MagCellect Mouse CD8^+^ T Cell Isolation Kit, R&D systems) T cells. Ninety six-well Immobilon-P membrane plates (MultiScreen-IP Filter Plate, MilliporeSigma, Burlington, MA, USA) were coated with rat anti-mouse gamma interferon (IFN-γ) antibody (clone AN18, BD Biosciences, Franklin Lakes, NJ, USA) at 2 μg/mL overnight at 4 °C. Plates were washed and blocked for 2 h in culture media. Then, isolated T cells were co-cultured with syngeneic splenocytes as antigen presenting cells (500,000 cells/well) and NP peptides (NR-18976, BEI Resources) in a total of 200 μL for 18 h at 37 °C in CO_2_ incubator. Plates were washed and the released IFN-γ was detected using a biotin-labelled anti-mouse IFN-γ (Biolegend, San Diego, CA, USA) followed by streptavidin conjugated with alkaline phosphatase (AP) (Jackson ImmunoResearch, West Grove, PA, USA). Following washing, plates were developed using Vector Blue Substrate Kit III (Vector Laboratories, Burlingame, CA, USA) and counted using an Immunospot Reader Series 2A with Immunospot software (Cellular Technology, Cleveland, OH, USA). Data were calculated by subtracting background values and were presented as spots per million CD4^+^/CD8^+^ T cells. Data represents samples from 3–5 individual mice.

B cell ELISpot assays were performed using single cell suspensions from mouse bone marrow [25]. Cells from virus-infected or mock-infected mice were harvested and processed in DMEM supplemented with 8% FBS. Plates were coated with Cal HA (NR-15749, BEI Resources) in PBS overnight at 4 °C. Plates were washed and blocked for 2 h in culture media. Bone marrow cells were plated and incubated for 18 h. Plates were washed and incubated with an anti-IgG-AP conjugate. The number of HA-specific IgG^+^ B cells were enumerated and reported as numbers per million bone marrow cells. 

### 2.7. Challenge Experiments

C57BL/6 mice were infected with wt or mutant Cal viruses at a dose of 100 pfu intranasally and allowed to sufficiently recover from primary infection in order to assess memory responses. At 84 days post primary infection, mice were challenged with wt Cal (10,000 pfu) or PR8 (500 pfu) and survival and weight loss were assessed for 15 days.

### 2.8. Statistical Analysis

Statistical analysis for all experiments was performed using one-way ANOVA followed by Tukey’s multiple comparison test (Prism 8, GraphPad Software, San Diego, CA, USA). A *p*-value of <0.05 was considered statistically significant.

## 3. Results

### 3.1. Shutoff Active NS1 Attenuate Viral Pathogenicity in Mice 

**Figure 1:**
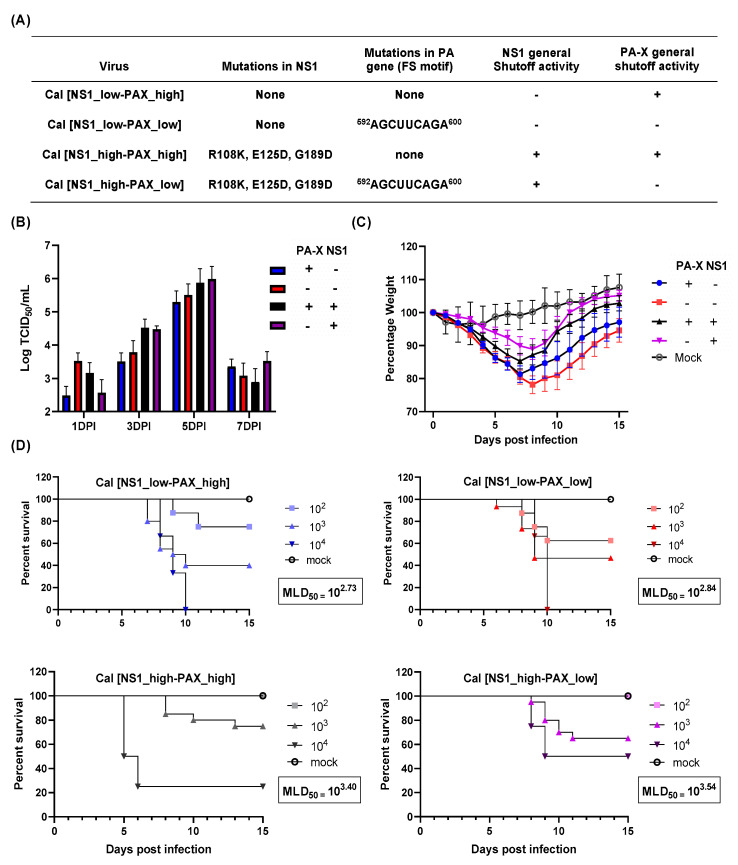
Virus growth and pathogenicity of mutant Cal viruses. (**A**) Mutations in the viruses analyzed in this study. (**B**,**C**) C57BL/6 mice were infected with the wt and mutant Cal viruses at a dose of 10^3^ pfu intranasally. (**B**) Virus titer in the lung was measured at 1, 3, 5 and 7 dpi (*n* = 4). (**C**) Body weight of infected mice was measured for 15 days (*n* = 12). (**D**) MLD_50_ was measured by infecting mice with 10^2^, 10^3^, or 10^4^ pfu of each virus. Survival of infected mice was measured daily until 15 dpi (*n* = 5).

To analyze the contribution of PA-X and NS1 to viral pathogenicity in vivo, we infected C57BL/6J mice with recombinant pH1N1 A/California/04/09 viruses with altered NS1 and/or PA-X activity (Figure 1A) [4]. First, we determined virus growth in infected mouse lungs. Lung virus titers ranged from 10^2.5^–10^3.5^ TCID_50_/_mL_ when determined 1 dpi. Although it was not statistically significant, the Cal[NS1_low-PAX_low] outgrew the other viruses, expressing shutoff protein(s) at 1 dpi, similar to what we observed in cultured MDCK cells [4]. All the viruses grew well in mice, reaching the peak titer at 5 dpi and then declining at 7 dpi (Figure 1B). No virus was detected in lungs at 9 dpi. Although it was not statistically significant, we detected higher virus titer in mice infected with viruses expressing shutoff active NS1 (Cal[NS1_high-PAX_low] and Cal[NS1_high-PAX_high]) on both 3 and 5 dpi. Interestingly, even though shutoff active NS1 viruses grew to a higher titer in lungs, mice infected with these viruses showed a decreased morbidity and mortality, as seen in the MLD_50_ and weight loss values (Figure 1C,D). Expression of shutoff active NS1 reduced MLD_50_ by 5–7 fold when compared to the corresponding viruses (PA-X low or high) (Figure 1D). Body weight loss of infected mice was consistent with the value of the MLD_50_ (Figure 1C). In contrast, we did not detect substantial changes in MLD_50_ based on PA-X expression (Figure 1D). MLD_50_ of Cal[NS1_low-PAX_high] was almost equivalent to the MLD_50_ of Cal[NS1_low-PAX_low]. These results indicate that the lung virus titer does not directly correlate to IAV pathogenicity, and suggest that host responses to viral infection strongly affect viral pathogenicity. 

### 3.2. Both Shutoff Active NS1 and PA-X Limit Early Inflammatory Responses and Immune Cell Recruitment in the Lung 

**Figure 2:**
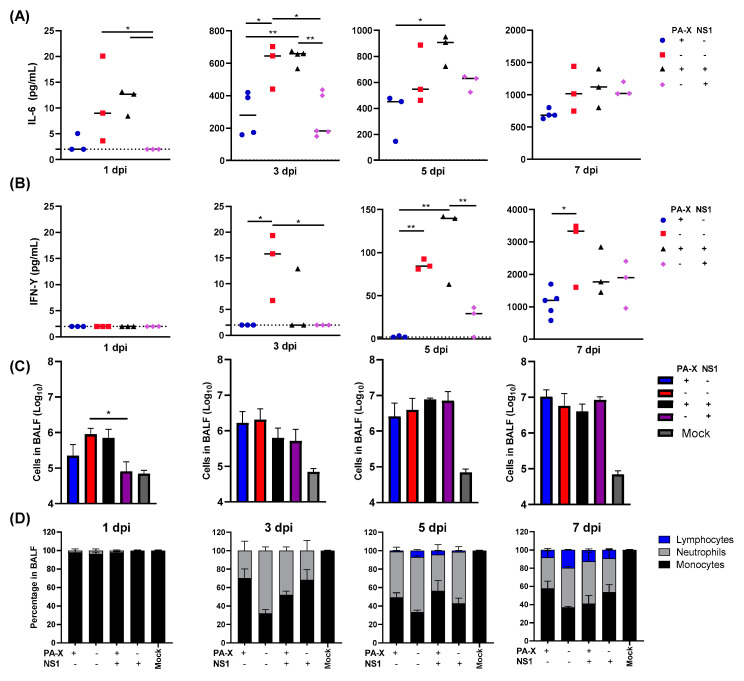
Kinetic analysis of cytokine production and infiltrating leukocytes in the lung following viral infection. C57BL/6 mice were infected with the viruses at a dose of 10^3^ pfu intranasally. (**A**,**B**) At 1, 3, 5 or 7 dpi, mice from each group were humanely sacrificed, and soluble IL-6 or IFN-γ in BALF was quantified. Dotted lines represent the limit of detection (2 pg/mL). (**C**,**D**) Infiltrating cells in BALF were enumerated and classified (*n* = 3–6). * *p* value < 0.05, ** *p* value < 0.01.

IAV-infected airway and alveolar epithelial cells undergo apoptosis or necrosis and trigger inflammatory responses to promote immune cell infiltration into the lung [26]. Cytokines released from infected epithelial cells also affect the early inflammatory response and infiltration of neutrophils and lymphocytes to the lungs. Our previous study on the same shutoff mutant viruses showed that shutoff active NS1 specifically suppresses cytokine genes in human airway cells [4]. We therefore determined the host cytokine response and immune cell recruitment to the lung upon virus infection in vivo. Mice were infected with the wt or mutant Cal viruses, and bronchoalveolar lavage fluid (BALF) was collected at 1, 3, 5, and 7 dpi. The IL-6 and IFN-γ in BALF was then directly quantitated by ELISA. As anticipated, the virus that was deficient in general shutoff activity (Cal[NS1_low-PAX_low]) strongly induced IL-6 and IFN-γ throughout the time points tested (Figure 2A,B). Although cytokine levels cannot be directly compared to each other in vivo because of the difference of virus titer in lungs (Figure 1B), we detected a lower level of IL-6 and IFN-γ in Cal[NS1_high-PAX_low]-infected mice than Cal[NS1_low-PAX_low]-infected mice, even though the virus grew to a higher titer in the respiratory tract. Compared to the Cal[NS1_high-PAX_low] virus, infection with Cal[NS1_high-PAX_high] induced more IL-6 and IFN-γ, even though it grew to a similar level and expresses shutoff active NS1. This likely reflects the fact that PA-X also targets the viral NS gene if NS1 has a shutoff activity, and reduces expression of NS1 [4]. The impact of shutoff active NS1 is less evident with this virus, possibly due to its reduced expression. Overall, these results suggest that shutoff active NS1 plays a key role in suppressing the release of IL-6 and IFN-γ in the airway of infected mice. 

We further determined immune cell infiltration by quantifying cells in BALF. Lymphocytes, neutrophils and monocytes were identified by their morphology after cytospin and staining. At early time points after infection on days 1 and 3, infiltration of neutrophil and lymphocytes was most strongly induced in mice infected with IAV having no shutoff activity (Figure 2C). At 1 dpi, the level of immune cell infiltration correlated with the virus titers in the lung. Interestingly, we detected reduced levels of cell infiltration in mice infected with IAV expressing shutoff active NS1 at 3 dpi, even though the lung virus titers are higher (Figure 1B and Figure 2C). Both shutoff active NS1 and PA-X contributed to suppression of neutrophil and lymphocyte recruitment to the lung, with a roughly 2-fold decrease in neutrophils at 3 dpi and a 2–5-fold reduction in lymphocytes at 5 and 7 dpi when compared to the virus with no shutoff activity (Figure 2C,D). These data show that expression of either PA-X or shutoff active NS1 is sufficient to reduce early cytokine production in the lung and to reduce effector cell presence in the tissue at early time points. 

### 3.3. NS1 Reduces the Immune Cell Infiltration and Extended Neutrophil Recruitment into Lung Tissue Following Acute Infection

**Figure 3:**
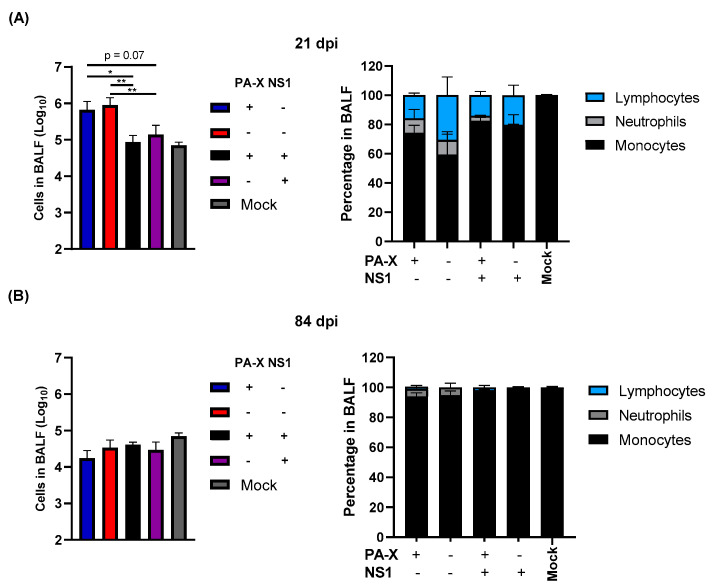
Shutoff active NS1 limits prolonged immune cell infiltration and prevents the extended recruitment of neutrophils to lung tissue. C57BL/6 mice were infected with the viruses at a dose of 10^3^ pfu intranasally. At 21 (**A**) and 84 (**B**) dpi, three mice from each group were humanely sacrificed. Magnitude and type of immune cell infiltrates in BALF were determined. (*n* = 3–7). * *p* value < 0.05, ** *p* value < 0.01.

We next determined the resolution of infiltrated immune cells in infected mouse lungs at 21 and 84 dpi. At 21 dpi, large amounts of immune cells remained within the lung of mice infected with the virus without shutoff activity, suggesting prolonged stimulation of the immune response (Figure 3A). Interestingly, PA-X could not rapidly clear infiltrated immune cells in the lung, although the ratio of lymphocytes to total cells is reduced compared to Cal[NS1_low-PAX_low] infected mice (Figure 3A). Similar amounts of neutrophils remained in BALF, suggesting that inflammatory cells are still being actively recruited into the lung tissue. In contrast, mice infected with IAV expressing shutoff active NS1 rapidly cleared infiltrating immune cells by 21 dpi. By 84 dpi, most of the infiltrated cells were cleared in all cases, but a higher ratio of neutrophils was still detected in lungs infected with Cal[NS1_low-PAX_low] or Cal [NS1_low-PAX_high] viruses, suggesting that the inflammatory response remains for a prolonged period in mice infected with viruses without shutoff active NS1. These data suggest that shutoff active NS1 plays a major role in the resolution of primary infection. 

### 3.4. Effect of Shutoff Proteins in Generating IAV-Specific T Cells and Serum Antibodies

Differences in cellular infiltration into the lung likely affect the generation of IAV-specific T cell and B cell responses. To examine the generation of IAV-specific immune responses, we first quantified IAV-specific T cell generation in the mediastinal lymph node (MLNs) and spleen at 7 and 21 dpi. IAV-specific CD4^+^ and CD8^+^ T cells were quantified by IFN-γ ELISpot following stimulation with NP peptide [24,27]. 

**Figure 4:**
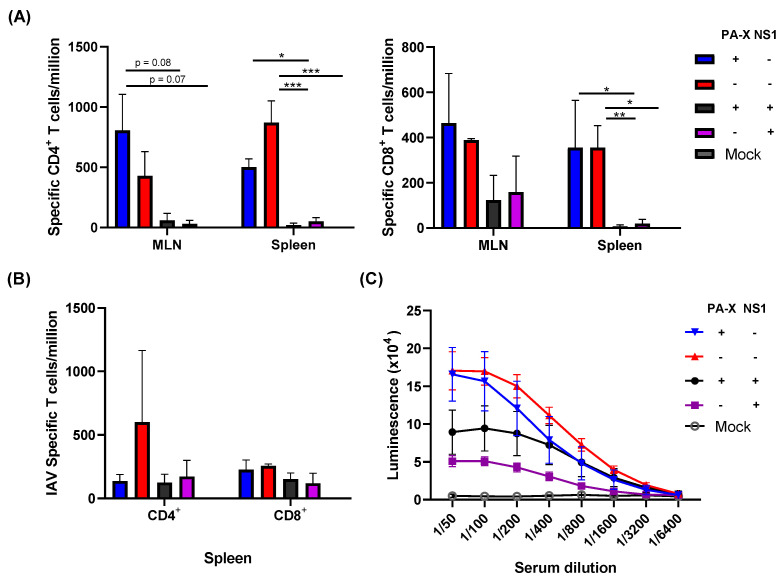
Early IAV-specific T and B cell responses. C57BL/6 mice were infected with the viruses at a dose of 10^3^ pfu intranasally. At 7 (**A**) or 21 (**B**) dpi, mice from each group were humanely sacrificed and CD4^+^ or CD 8^+^ T cells were isolated from the MLN or spleen. Influenza-specific T cells were quantified following the stimulation of negatively selected T cells with NP peptide and detecting NP-specific T cells by IFN-γ ELISpot. (**C**) The serum of infected mice were collected at 21 dpi, and HA-specific antibody was measured by ELISA using purified HA (*n* = 3–4). * *p* value < 0.05, ** *p* value < 0.01, *** *p* value < 0.001.

Despite larger titers of viruses recovered from the lung, infection with viruses expressing shutoff active NS1 (Cal[NS1_high-PAX_low] or Cal[NS1_high-PAX_high]) resulted in significantly fewer IAV-specific CD4^+^ and CD8^+^ T cells in the MLN and spleen at 7 dpi (Figure 1B and Figure 4A). The effect of PA-X in suppressing T cell responses was less clear than the effects of NS1, although slightly fewer IAV-specific CD4^+^ T cells were detected in the spleen of Cal[NS1_low-PAX_high] infected mice. To determine whether the difference in specific T cell generation was due to a delay in T cell generation during the acute phase of infection, we quantified IAV-specific T cells in the spleen at 21 dpi. Similar to what we observed at 7 dpi, only the virus without shutoff proteins generated and maintained a high level of IAV-specific CD4^+^ T cells in the spleen (Figure 4B). The suppressive effects of NS1 and PA-X were less clear for CD8^+^ T cells at 21 dpi. In addition to examining the T cell response, we also determined serum IgG levels against IAV HA protein. Similar to specific T cell generation, shutoff active NS1 reduced IgG HA^+^ serum antibody levels at 21 dpi (Figure 4C). The ELISA binding curve also suggests a weak affinity for the HA antigen, which could have implications on B cell fate determination [28]. Taken together, these data clearly show that active NS1 strongly affects the generation of early IAV-specific T and B cell responses.

### 3.5. Shutoff Active NS1 Limits IAV-Specific Adaptive Immunity 

**Figure 5:**
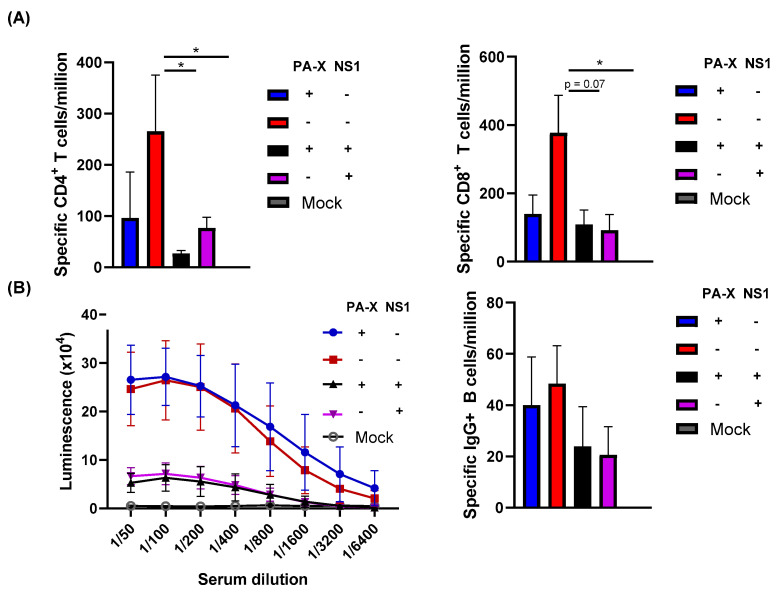
Effect of shutoff proteins in the generation of specific T and B cell responses. C57BL/6 mice were infected with the viruses at a dose of 10^3^ pfu intranasally. (**A**) At 84 dpi, mice from each group were humanely sacrificed and IAV-specific CD4^+^ or CD8^+^ cells in the spleen were quantified using IFN-γ ELISpot. (**B**) B cell responses were analyzed by quantifying HA-specific serum IgG using ELISA and HA-specific plasma cells retained in the bone marrow by ELISpot (*n* = 3–6). * *p* value < 0.05.

While NS1 clearly affected early adaptive immune responses to acute infection, it is unclear whether this could affect long-lasting adaptive immunity. To determine if NS1 and PA-X affect the maintenance of IAV-specific adaptive immune responses, we infected mice with the viruses and measured B and T cell responses at 84 dpi. IAV-specific T cells in the spleen were analyzed by T cell ELISpot. At this late time point, both IAV-specific CD4^+^ and CD8^+^ T cell levels were significantly reduced by either NS1 or PA-X shutoff activities, suggesting that both proteins can suppress the generation of memory T cells (Figure 5A). Shutoff active NS1 also effectively suppressed the generation of serum antibody against HA, correlating to the reduced levels of HA-specific B cells in bone marrow (Figure 5B). PA-X was less effective in suppressing B cells compared to CD4^+^ or CD8^+^ T cells, which reflected the level of serum antibody against HA. These results indicate a functional difference between NS1 and PA-X in regulating host-acquired immune responses.

### 3.6. NS1, but Not PA-X, Prevents the Generation of Protective Immunity against Heterologous Virus Challenge 

**Figure 6:**
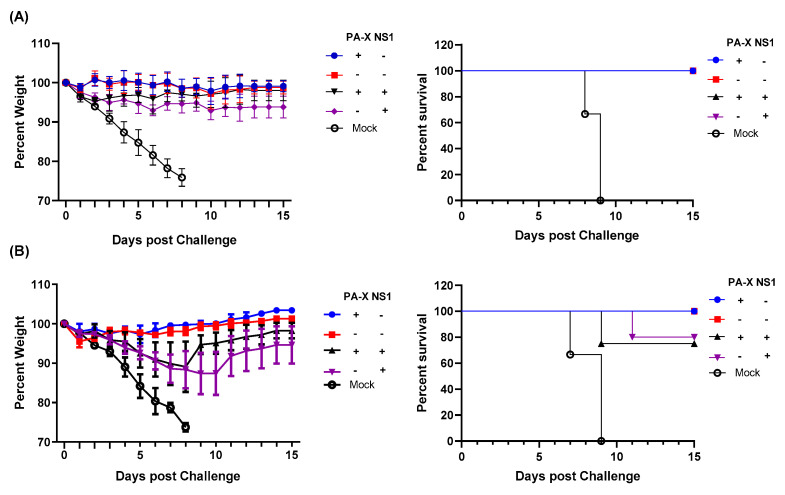
Shutoff active NS1 suppresses generation of protective immunity against heterologous virus challenge. C57BL/6 mice were infected with wt or mutant Cal viruses at a dose of 100 pfu intranasally. At 84 dpi, mice from each group were challenged with 10,000 pfu of wild type Cal (**A**) or 500 pfu of PR8 (**B**). Body weight and survival rate were monitored for 15 days after the challenge (*n* = 4–5).

Finally, we assessed protective immunity induced by the mutant viruses against a homologous or a heterologous challenge. We first infected with wt or mutant viruses, and 84 days later, challenged the recovered mice with a lethal dose (10 × MLD_50_) of wt Cal or A/Puerto Rico/8/34 (H1N1) (PR8). Body weight loss and survival rate were monitored for 15 days. Initial infection with any of the four different viruses produced sufficient immunity to protect the mice from a homologous virus challenge (Figure 6A). When challenged with heterologous virus PR8, mice initially infected with Cal[NS1_low-PAX_low] were protected from the challenge. Similarly, mice infected with the virus expressing only PA-X (Cal[NS1_low-PAX_high]) were protected from PR8. In contrast, mice that were first infected with the virus expressing shutoff active NS1 were not completely protected from the challenge with the heterologous virus. These mice lost 10–15% of their body weight, and 20–25% of mice did not survive (Figure 6B). These results demonstrate the impact of shutoff active NS1 in suppressing the host-acquired immune response required for protection from repeated infection.

## 4. Discussion

The ability of viral pathogens to counteract host innate and acquired immune responses is a major determinant of pathogenicity. IAV antagonizes host antiviral and immune responses by various approaches mainly mediated by two accessory proteins, NS1 and PA-X. Both proteins induce general shutoff, but their specificity of target genes is different. Our transcriptomic data of infected A549 cells indicated that PA-X has a broad target range, while shutoff active NS1 specifically limits host genes involved in innate and acquired immune responses [4]. While both proteins contribute to antagonizing host innate responses, the variation in target specificity may have different effects on the development of acquired immune responses in the host. In this study, we analyzed the effect of shutoff proteins on the host acquired immune response using a mouse model and revealed a major impact of NS1 shutoff activity in suppressing the generation of IAV-specific B and T cells and protective immunity.

Initial infection of respiratory cells with the virus triggers reactions to induce cytokine and chemokine release, which attract immune cells to the site of infection. Our transcriptomic analysis of human airway cells infected with the shutoff mutant viruses used in this study showed NS1-specific suppression of key pro-inflammatory cytokine genes such as IL-1 and IL-6. Shutoff active NS1 also suppressed the expression of CCL5, CXCL10, and CXCL11, which are chemoattractants for monocytes and T cells [29]. This function is likely reflected in the delayed infiltration of immune cells in the respiratory tract of mice, even though higher levels of viruses exist in the lung (Figure 1B and Figure 2C). In addition to delayed infiltration, shutoff active NS1 contributed to reduced duration of cellular recruitment to the lung following acute infection (Figure 3A,B). Significantly higher numbers of infiltrating cells remained at 21 dpi in mice infected with Cal[NS1_low-PAX_low] or Cal[NS1_low-PAX_high], in contrast to the mice infected with the viruses expressing shutoff active NS1 (Figure 3A). These results strongly suggest that NS1 prevents continuous stimulation of the immune response in the lungs through its shutoff activity. Consistent with this, early development of IAV-specific CD4^+^ and CD8^+^ T cells was restricted in mice infected with viruses expressing shutoff active NS1 (Figure 4A). This limited generation of IAV-specific T cells may also be caused by specific suppression of MHC I molecules by shutoff active NS1 [4]. 

This effect on IAV-specific T cell generation may also account for viral pathogenicity. We observed the attenuation of viral pathogenicity with NS1 shutoff activity (Figure 1C,D), which could be explained by the reduced presentation of IAV antigens and T cell-mediated cell and tissue damage. Antiviral T cells are crucial for the elimination of various respiratory viruses, but have been shown to contribute significantly to immunopathological tissue damage during and after viral clearance [30,31,32]. Infiltrated neutrophils also contribute to disease-associated tissue damage [33], and our data indicate reduced long-term neutrophil infiltration in mice infected with shutoff active NS1-expressing viruses (Figure 3). During primary infection, neutrophils migrate to the inflamed tissue and produce neutrophil extracellular traps in the affected tissue. This immune response leads to epithelial cell damage and the production of cellular debris, which furthers inflammatory responses and can cause impaired lung function [33]. A lack of shutoff active NS1 resulted in prolonged infiltration of neutrophils, even at 84 dpi (Figure 3B), stressing the impact of NS1 shutoff activity in viral pathogenesis. 

In addition to the T cell response, shutoff active NS1 strongly limited the production of specific serum antibodies and long-lived plasma cells in the bone marrow (Figure 5B). Even with the Cal[NS1_high-PAX_high] virus, which expresses less shutoff active NS1 protein compared to the Cal[NS1_high-PAX_low] virus, the production of specific serum antibodies was significantly reduced. Generation of antigen specific B cells relies on the presence of corresponding antigen-specific CD4^+^ T cells [34]. The serum antibody titers in infected mice correlated well to the CD4^+^ T cell responses at 7 dpi, suggesting that early stage antigen presentation and the generation of specific CD4^+^ T cells determine B cell responses, which are maintained at late time points (Figure 4A and Figure 5B). B cell memory is known to play a significant role in neutralizing viruses, including in response to reinfection [35]. Despite lower titers, mice first challenged with viruses expressing active NS1 showed only marginal weight loss following a lethal homologous challenge, suggesting that this level of antibody is still effective in protecting against homologous virus reinfection (Figure 6A). In the case of a heterologous virus challenge, a reduced antibody level seems to be ineffective in completely blocking subsequent viral infection. Because no major difference was detected in the level of specific CD4^+^ and CD8^+^ cells at the time of challenge (Figure 5A), the presence of high levels of antibody induced by Cal[NS1_low-PAX_high] is likely to be sufficient for protection from PR8 virus. The impact of PA-X in suppressing acquired immunity was less evident compared to shutoff active NS1, even though PA-X is more potent in inducing general host shutoff [4]. However, PA-X still slowed the infiltration of immune cells (Figure 2C,D), possibly due to reduced cytokine and chemokine release. This effect may also contribute to the slowed development of immunity during acute infection.

Not surprisingly, infection with the virus lacking shutoff activity induced strong early cytokine responses, long-lived increases in IAV-specific CD4^+^, CD8^+^, and serum antibodies, as well as protection against both homologous and heterologous challenges. These results indicate that IAV without shutoff activity induces strong immunity against the virus. The shutoff activity of NS1 and PA-X of current live attenuated influenza vaccines (LAIV) has not been determined in detail, but considering the strong impact on the outcome of immunity found in this study, it is possible that modification of NS1 shutoff activity strongly improves the potential of the LAIV and generates robust, long lasting, cross-reactive immunity.

The functional differences of PA-X and NS1 shutoff activities in generating protective immunity could be an important factor for IAV adaptation to human hosts. Most of the circulating human IAV strains express shutoff active NS1 and weakly active PA-X. The key NS1 residues responsible for CPSF30 interaction and shutoff activity are less conserved among animal isolates. Comparison of PA-X shutoff activity of human and animal isolates also indicate species-specific differences. PA-X of avian viruses and 2009 pH1N1 containing the PA segment originated from avian viruses is highly active compared to those from human viruses [16]. The presence of highly active PA-X and NS1 is less effective in antagonizing host immune responses because PA-X also targets and degrades viral NS genes in the presence of shutoff active NS1 [4]. Therefore, fine tuning of its NS1 and PA-X shutoff activities might occur for the Cal virus to be effectively circulated within the human population.

## 5. Conclusions

In conclusion, shutoff active NS1, which is conserved among human IAVs except 2009pH1N1, efficiently limits the generation of IAV-specific B and T cells and long-lasting immunity to protect against subsequent infection. While PA-X has stronger shutoff activity on general gene expression, it is less effective in regulating the acquired immune response.

## Data Availability

The data presented in this study are available on request from the corresponding author.
